# Association of DNA biosynthesis with planting value enhancement in hydroprimed maize seeds

**DOI:** 10.1016/j.sjbs.2021.02.068

**Published:** 2021-03-01

**Authors:** Heena Rasool Mir, Shiv Kumar Yadav, Sezai Ercisli, Asma A. Al-Huqail, Dina A. Soliman, Manzer H. Siddiqui, Saleh Alansi, Sangita Yadav

**Affiliations:** a Division of Seed Science and Technology, ICAR- Indian Agricultural Research Institute, New Delhi 110012, India; b Department of Horticulture, Ataturk University, Erzurum 25240, Turkey; c Department of Botany and Microbiology, College of Science, King Saud University, Riyadh 11451, Saudi Arabia

**Keywords:** Cell cycle, DNA replication, Flow cytometry, Hydropriming, Maize, Planting value, Seed enhancement

## Abstract

Inadequate plant stand establishment due to insufficient germination is an important bottleneck in achieving the potential yields, specifically under uncertain growing conditions. Hydropriming has been publicized as a useful tool to alleviate the stress-induced consequences. Association of DNA biosynthesis in hydroprimed seeds of maize; hybrid, PEHM 5 and its parental lines (CM150 and CM151) was studied. Seeds were hydroprimed at 25 °C for 30 h and half of them were surface dried while the other half were redried back to the original moisture contents. The treated and untreated seeds were evaluated for; germination test, mean germination time, vigour index and DNA levels in embryos of fully matured seeds. Both the treatment strategies significantly enhanced the planting value of maize seeds. Vigour index I revealed significant correlation with G2/G1 ratio whereas significant negative correlation between G2/G1 ratio and mean germination time was observed. Large amounts of 2C DNA signals in flow cytometric analysis divulged that most cells might had arrested in the cell cycle at the pre synthetic G1 phase of nuclear division. Augmentation of 4C signal in the embryonic region was noticed after imbibition that could be ascribed to cells entering the synthetic phase of nuclear division. The embryonic cells showed increased 4C:2C ratios after 30 h of imbibition. Apparently, DNA synthesis preceded germination. In dry seeds, DNA histograms revealed both a 2C signal and a considerable 4C peak. A priming period of 30 h in distilled water considerably enhanced the rate and uniformity of germination in both surface dried and redried treatment strategies. Upon priming, the ratio of 4C:2C increased during the 30 h priming period, though the level in case of redried seeds did not reach the level obtained after hydration in water without drying back. However, the 4C: 2C ratio was constant after redrying the seeds to the original moisture content, indicating that the chromosomal material in the embryonic cells had stably ceased cell cycle activity at the G2 phase. The present results indicate that the beneficial effects of priming on seedling performance could be associated with the action of replicative DNA synthesis processes prior to germination.

## 1 Introduction

Presowing treatments like hydropriming involves seed imbibition with adequate water content for the activation of pre-germinative metabolic processes without radicle protrusion. After imbibition, seeds are dried to their original moisture content. The primed seeds exhibit improved seed performance and facilitate rapid and synchronised rates of radicle emergence (Pedriniet al., 2020). The post-priming improvement in seed performance has been explained in terms of increased activity of enzymes involved in reserve mobilisation (Tounekti, et al., 2020), repair processes of DNA and proteins (Varier et al., 2010), and by enhancing various germination promoting metabolites (Hussain et al., 2015) leading to more favourable cellular and metabolic balance at the time of germination.

The cell cycle is a synchronised series of events that allows the cell duplication of its DNA and segregation into daughter cells. It consists of DNA replication (S phase) and mitotic cell division (M phase), preceded by gap phases, G_1_ and G_2_. G_1_ is the first gap phase, wherein the nucleus has 2C DNA (C = DNA content of a holoploid genome with chromosome number *n*) and is the entry point leading to cell cycle advancement after a non-proliferative period. The cell replicates in S phase, which cause duplication of DNA from 2C to 4C. Moreover, transition from G_1_ to S is the main point of the cross talk between activation of cell cycle and environmental signals. Considering the fact that seed germination is the resumption of cell division and growth after a long period of quiescent in dry seeds, triggering of the G_1_ to S switch could probably play important role in regulation of early seedling development triggered due to these presowing treatments.

Several studies have documented that seed priming causes an enhancement in the nuclear DNA content from 2C to 4C in radicle meristem cells, which signify advancement of G_1_phase to the S or G_2_ phases of the cell cycle (Vázquez-Ramos and Sánchez, 2003; (Vázquez-Ramos and Sánchez, 2007) Alžběta et al., 2020). Mean germination time was significantly correlated with increase in 4C nuclei in pepper (*Capsicum annum* L.) and tomato (*Lycopersicon esculentum* Mill.) primed seeds (Gurusinghe et al., 1999a, Gurusinghe et al., 1999b). It was also reported that increase in 4C nuclear DNA occurs during priming of *Beta vulgaris* L. seeds, without cell division (Redfearn and Osborne, 1997). Therefore, it has been anticipated that seed germination was positively linearly correlated with increase in DNA synthesis and efficiency of a priming treatment (Özbingöl et al., 1999). Nevertheless, the effect of a certain pre-sowing treatment on advancement of DNA synthesis differed among seed lots and was dependent on osmotic potential used and duration of the treatment (Van Pijlen et al., 1996). During seedling growth of primed seeds, the increase in proportion of the 4C DNA indicates the entry of cells into G2 phase, which precedes M phase. As a result, the ratio of 4C/2C has been considered as a marker for the advancement of seed germination (Vázquez-Ramos and Sánchez, 2003; Kolano et al., 2008). However, for the cells in seeds undergoing endoreduplication recommended to be negatively correlated with genome size, instead (Rewers and Sliwinska, 2012). Cell cycle activity is important event and required for the seeds to grow and develop into a plant. But its involvement in the early events that initiate seedling emergence and development is relatively less explored in primed maize seeds. Therefore, the present studies were undertaken to investigate the effect of presowing hydropriming treatments on embryo cell cycle in the seeds of maize hybrid and parental line by means of flow cytometry.

## 2 Materials and methods

To appraise the effect of priming treatments on cell cycle activity, genetically pure and fresh seed lots of PEHM 5 hybrid and its parental lines; Female – CM 150, Male - CM 151 were obtained from Seed Production Unit, ICAR-Indian Agricultural Research Institute, New Delhi. Seeds of these three genotypes were subjected to hydropriming treatment with distilled water for 30 h at 25 °C. Hydroprimed seeds of each genotype were divided in two equal portions. The first portion (T_1_) was surface dried only (surface of seeds was dried with blotting papers and used immediately), second portion (T_2_) was redried (the seeds were removed from the distilled water and redried back to the original moisture content under the shade at room temperature at 25 ± 2 °C) and untreated seeds (T_0_) were used as control. All these seed lots, thus, obtained were used to assess seed quality parameters namely; germination test, mean germination time, vigour index and DNA levels in embryos of fully matured seeds. The flow cytometric studies with 5 replications were carried out at the laboratory of Division of Plant Improvement, Indian Grasslands and Forage Research Institute (IGFRI), Jhansi, India whereas, seed quality was assessed in 3 replications at the laboratories of Division of Seed Science and Technology, ICAR-IARI, New Delhi.

Standard method (ISTA, 2013), with minor modifications was adapted to determine the seed germination by putting 50 seeds of each genotype in 3 replications in moist paper towel at 25 °C in the walk-in-germinator. The seeds that were rolled between two layers were evaluated for first count on 4th day and final count was recorded on 7th day. On the day of final count, numbers of normal seedlings were counted and percent germination was calculated.

On the day of final count for the calculation of germination percentage 10 normal seedlings from each replication were randomly picked up to measure the root and shoot lengths of seedling. The mean values were used for computing the vigour indices by using the following formula (Abdul-Baki and Anderson, 1973):

Vigour Index I = Germination (%) × Total seedling length (cm)

The seeds that were put for germination were counted daily till 7th day for calculation of mean germination time (MGT). The formula ($n\times \frac{d}{N}$) was used for the estimation of MGT (Ellis and Roberts, 1980). In formula, ‘n, d and N’ represents the number of seeds germinated daily, days since the initiation of test and total seeds germinated at the end of test, respectively.

**Fig. 1:**
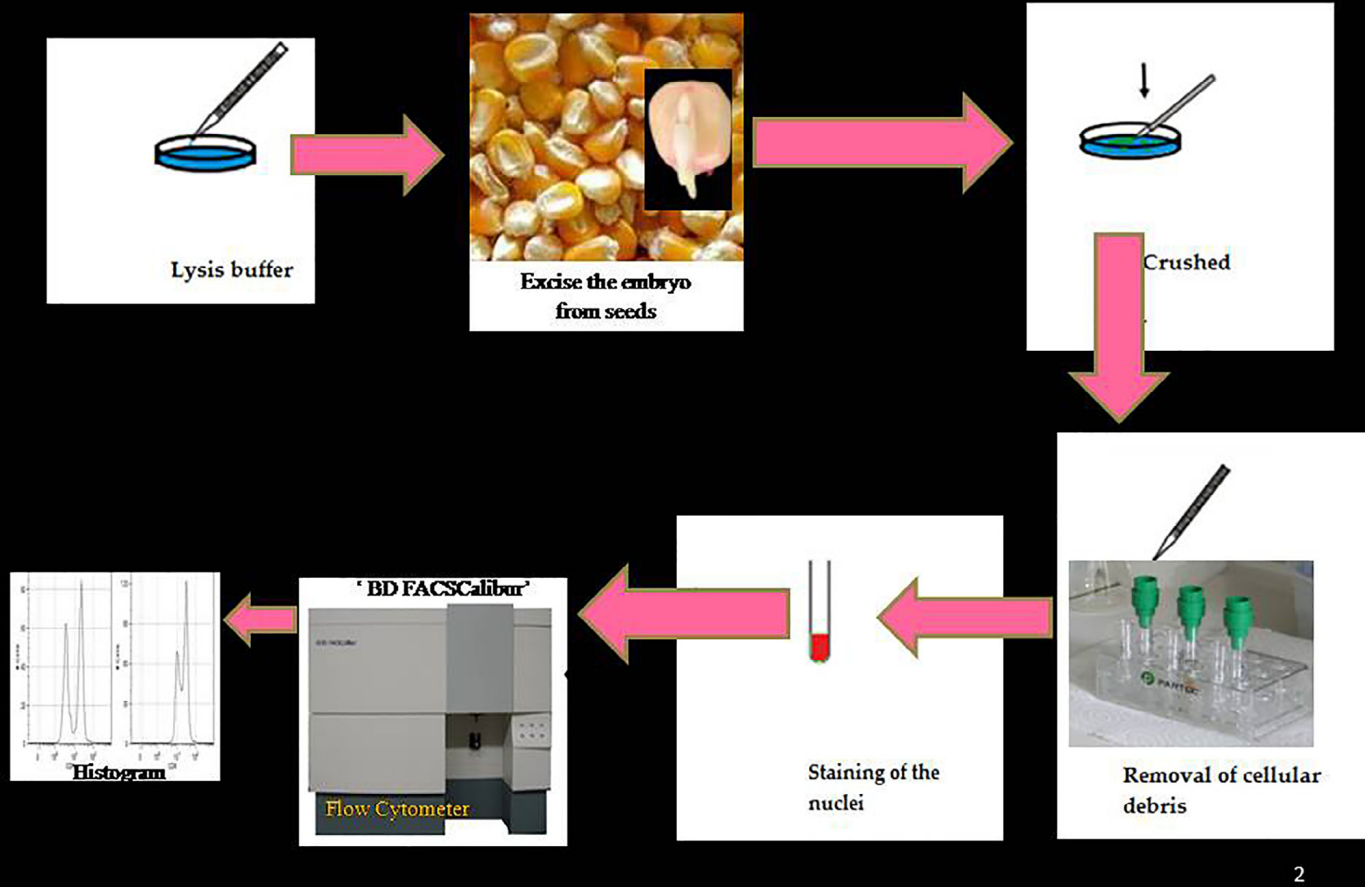
Steps for the estimation of nuclear DNA content in maize seeds by flow cytometry.

Nuclear samples for flow cytometric analysis were prepared according to reported procedure with minor modifications, “The individual seeds were used and embryo was chopped with a sharp razor blade in a Petri dish containing 0.5 ml nuclei isolation buffer (Chemunex, Moisons-Alfort, France), supplemented with 4, 6-diamidino-2-phenylindole (DAPI). After chopping, 1 ml of buffer was added and the mixture was filtered through a 30-µm mesh nylon filter. DNA content was measured after about half an hour in a Partec CA II flow cytometer (Partec, Munster, Germany). In each sample, about 5000 nuclei were analysed. The Partec DPAC V2.1 computer programme was used for peak analysis” (Galbraith et al., 1983). For each of the seed sample, 5 replications were analysed as per the procedure depicted (Fig. 1).

Before analysis of variance using factorial completely randomized design, percentage data were transformed to arc sine values using the formula in Microsoft excel (fx =+ASIN (SQRT ((%Value)/100))*180/3.14). For performing the Student’s *t*-test (McDonald, 2008) on the G_2_/G_1_ ratios, angular transformation of data was done for independent samples. The correlation coefficient among the various parameters was also calculated using SPSS for Windows, Version 10.0. Chicago, SPSS Inc.

## 3 Results

The initial germination recorded was 85, 80 and 92 per cent in untreated seeds of Female, CM-150; Male, CM-151 and maize hybrid, PEHM-5, respectively (Table 1). The mean initial moisture content recorded was 10.3%, however 100 seed weight varied and it was 17.46, 18.35 and 17.84 g in female, male and hybrid seeds, respectively. The studies on effect of seed hydropriming with distilled water for 30 h at 25 °C revealed that the effectiveness of both the surface dried and redried treatments was remarkably different as compared to untreated (control) seeds. The influence of hydropriming on germination percentages among the genotypes was also found to be significant and it was significantly higher in surface dried seeds (92.3%) and redried seeds (91.7%) over control (85.7%). The enhancement in germination per cent of surface dried and redried seeds of CM 150 was by 11.8 and 9.4 per cent, that of CM 151 by 7.5 and 8.8 per cent and in PEHM 5, it was 4.3 and 3.3 per cent respectively, as compared to untreated seeds (Table 2).

**Table 1:** Initial status of seed quality parameters of different maize genotypes.

Genotype	100 seed weight (g)	Moisture (%)	Germination (%)
CM-150 (Female)	17.46	10.4	85.0
CM-151(Male)	18.35	10.2	80.0
PEHM-5 (Hybrid)	17.84	10.2	92.0
Mean	17.88	10.3	85.7
SEm (±)	0.26	0.07	3.48

**Table 2:** Effect of different hydropriming treatments on germination (%) of maize hybrid PEHM 5 and its parental line seeds.

Genotypes	Control	Primed (surface dried)	Primed (redried)	Mean (B)
CM 150	85.00 (67.63)*	95.00 (77.12)	93.00 (75.20)	91.00 (73.31)^a^
CM 151	80.00 (63.44)	86.00 (68.34)	87.00 (68.62)	84.33 (66.80)^b^
PEHM 5	92.00 (73.92)	96.00 (78.46)	95.00 (77.12)	94.33 (76.40)^c^
Mean (A)	85.67 (68.33)#^a^	92.33 (74.20)^b^	91.67 (73.92)^b^	
C.D. (p = 0.05) Genotype (A) Treatment (B) Interaction (A X B)		2.5 2.5 NS		

*Values in parenthesis are the arc-sine values. NS: Non-significant.

# Data with same alphabet don’t statistically differ significantly.

Mean germination time (MGT) significantly varied among the maize genotypes studied, treatment strategies and their interactions. The hybrid, PEHM-5 noticed significantly lower MGT (3.2d) than the female line (3.4d), however it was significantly higher (4.5d) in the male line than the other two genotypes (Table 3). MGT (d) was found significantly influenced in both surface dried and redried treatment strategies. MGT was significantly reduced to 2.8d in hydropriming (surface dried) treatment as compared to control (4.9d) that was significantly higher than that of the redired seeds (3.3d). The untreated seeds of male line, CM 151 took significantly highest mean time (5.4d) and surface dried seeds took significantly lowest mean time (2.3d) to germinate. The vigour index I (VII) was found paralleled to total seedling length, as it is the manifestation of germination percent and the total seedling length so the data of seedling length has not been given. The increase in VII of seeds ranged from 23.8 to 26.9 per cent in surface dried and 13.9–15.0 per cent redried seeds, respectively (Table 4). There were significant differences for VII among the genotypes and treatments, however non-significant interaction effect was noticed. The VII of male (3079) and female (3198) was found to be at par with each other but significantly lower than the hybrid, PEHM 5 (3553) seeds. The surface dried resulted in significantly higher VII (3630) than that of redired (3310) and control (2889) seeds.

**Table 3:** Effect of different hydropriming treatments on mean germination time (days) of maize hybrid PEHM 5 and its parental line seeds.

Genotypes	Control	Primed (surface dried)	Primed (redried)	Mean (B)
CM 150	4.67	2.50	3.07	3.41^a^
CM 151	5.43	3.70	4.27	4.47^b^
PEHM 5	4.70	2.33	2.60	3.21^c^
Mean (A)	4.93#^a^	2.84^b^	3.31^c^	
C.D. (p = 0.05) Genotype (A) Treatment (B) Interaction (A X B)		0.19 0.19 0.33		

# Data with same alphabet don’t statistically differ significantly.

**Table 4:** Effect of different hydropriming treatments on vigour index I of maize hybrid PEHM 5 and its parental line seeds.

Genotypes	Control	Primed (surface dried)	Primed (redried)	Mean (B)
CM 150	2809	3553	3231	3198^a^
CM 151	2704	3432	3101	3079^a^
PEHM 5	3155	3907	3597	3553^b^
Mean (A)	2889#^a^	3630^b^	3310^c^	
C.D. (p = 0.05) Genotype (A) Treatment (B) Interaction (A X B)		164 164 NS		

NS: Non-significant.

# Data with same alphabet don’t statistically differ significantly.

**Fig. 2:**
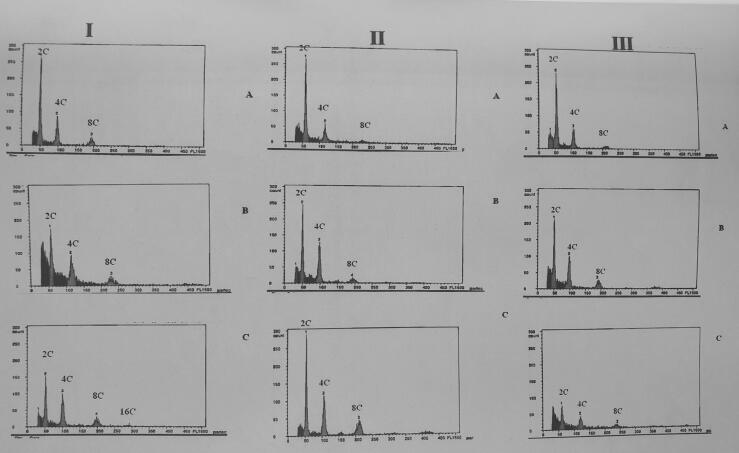
Histogram of flow cytometric analysis of nuclei from embryonic seed portion of (I) female line (CM 150); (II) male line (CM 151) and (III) hybrid (PEHM5) seeds. (A) untreated seeds (B) surface dried seeds and (C) redried seeds.

In the present study, hydropriming specific differences in nuclear DNA content profiles of seeds were studied by flow cytometry within each of the three genotypes (Fig. 2). Flow cytometric determination in chopped embryonic tissues of maize seeds showed that only 2C and 4C nuclei were present. The bigger peak at channel 50 indicated the 2C nuclei at G1 phase of nuclear division whereas, the smaller peak around channel 100 corresponds to 4C DNA at G2 phase. Similar profiles for mean percentage of cells in G2 phase were found in all three genotypes. Damaged nuclei as well as cell wall fragments smaller than 25 μm in size gave rise to background signals, which were predominantly at the lower channel numbers. The distribution of nuclei peaks, with DNA contents expressed as C values, in fully mature seeds were different for the different genotypes. Apart from these 2C signals, histograms of certain embryonic cells gave various combinations of C values in the range from 2C to 8C. For each genotype, the proportion of cells with a certain C value varied.

Subsequent to hydropriming, the proportions of 4C nuclei increased to 22.26% in surface dried treatment which was significantly higher than the untreated (14.75%) seeds. The proportions of 4C nuclei were 21.85% in male line, significantly higher than the 18.99 and 17.84% in hybrid and female line, respectively (Table 5). This could be due to increased cell cycle activity in preparation of cell division. In addition, all three genotypes showed enhanced G2/G1 ratio in seeds of both the hydropriming strategies as compared to control (Table 6). The G2/G1 ratio was significantly higher in surface dried (0.620) than the control (0.416) seeds. The G2/G1 ratio in female line and hybrid was at par with each other and it was significantly higher in male line (0.590) seeds. The germination, MGT and VII had highly significant positive correlations among them. The data showed correlations of seed quality traits with G2/G1 ratio (Table 7). G2/G1 ratio was found positively correlated with germination, however it showed significant positive (0.335) correlation with VII whereas, it exhibited significant negative (−0.309) correlation with MGT.

**Table 5:** Effect of different hydropriming treatments on percentage of cell numbers in the G2 phase (cell cycle) of maize hybrid PEHM 5 and its parental line seeds.

Genotypes	Control	Primed (surface dried)	Primed (redried)	Mean (B)
CM 150	15.07	19.62	18.84	17.84^a^
CM 151	15.05	24.80	25.70	21.85^b^
PEHM 5	14.13	22.38	20.47	18.99^a^
Mean (A)	14.75#^a^	22.26^b^	21.67^b^	
C.D. (p = 0.05) Genotype (A) Treatment (B) Interaction (A X B)		2.42 2.42 NS		

NS: Non-significant.

# Data with same alphabet don’t statistically differ significantly.

**Table 6:** Effect of different hydropriming treatments on G2/G1 ratio (cell cycle) of maize hybrid PEHM 5 and its parental line seeds.

Genotypes	Control	Primed (surface dried)	Primed (redried)	Mean (B)
CM 150	0.388	0.576	0.521	0.495^a^
CM 151	0.453	0.689	0.627	0.590^b^
PEHM 5	0.405	0.595	0.482	0.494^a^
Mean (A)	0.416#^a^	0.620^b^	0.543^c^	
C.D. (p = 0.05) Genotype (A) Treatment (B) Interaction (A X B)		0.11 0.14 NS		

NS: Non-significant.

# Data with same alphabet don’t statistically differ significantly.

**Table 7:** Coefficients of correlation between the G2/G1 ratio and seed quality traits of maize genotypes.

	Ger (%)	MGT	VII	G2/G1
Ger (%)	1.000	−0.783**	0.822**	0.204
MGT		1.000	−0.843**	−0.309*
VII			1.000	0.335*
G2/G1				1.000

**Significant (p = 0.01) and * Significant (p = 0.05).

## 4 Discussion

Significantly higher germination of primed seeds of all the genotypes might be attributed to the onset of early metabolic activities during hydration, leading to a physiological state that enables seed to the brink of radicle protrusion (Hussain et al., 2016). Among the genotypes, MGT was significantly reduced in surface dried hydropriming strategy. Being retained largely after non-drying of seeds, physiological advancement of primed seeds resulted in faster germination upon rehydration (McDonald, 2000). The rapid germination of hydroprimed seeds for surface dried and redried treatments appeared to be associated with uniform germination and early seedling growth. Moreover, seedling length and dry matter production was significantly increased in the case of hydroprimed seeds as compared to control, which could be attributed to reduced emergence time as evidenced by increased germination and vigour index. The germination and performance of low vigour seeds was reported to be better in hydropriming treatment, which was attributed to improved DNA repair and better maintenance of genome integrity (Bray and West, 2005).

Flow cytometry has made it possible to determine the DNA content in tissues of various plant species with high accuracy. The enhanced G2/G1 ratio could be associated with maintenance of DNA integrity and repair activity in hydroprimed seeds and thus the increased capacity of these seeds for rapid germination. Both the hydropriming treatments showed positive response with respect to all seed quality parameters, which could be probably due to early reactivation of DNA repair processes. This was not the case for hydropriming (redried) treatment, wherein even enhanced cell cycle activity was not significantly correlated with beneficial effect of treatment. An increase of 2–24 per cent was observed in cell cycle studies of sugar beet seeds, which were significantly higher than untreated seeds, indicating enhanced occurrence of DNA replication in such seeds (Redfearn and Osborne, 1997). The priming treatment increased G2/G1 ratio upon and it was faster when longer periods of hydration were applied (Sliwinska et al., 1999). The results of flow cytometric determination indicated that the cell cycle in hydroprimed seeds can have differential effect in different cultivars. Primed tomato embryos can advance from G1 to G2 phase, without undergoing the cell division process (Dawidowicz, 1997, De Castro et al., 2000), indicating that cell cycle activity can contribute to rapid and early germination due to seed priming. Some seed lots exhibited enhanced DNA content in the embryo with short soaking, but the effect was not evident in other lots. Similar results were also reported in tomato and pepper seeds (Gurusinghe et al., 1999a, Gurusinghe et al., 1999b). This could be attributed to different proportions of G2 cells upon priming in different seed lots. In some tomato seed lots, replicative DNA synthesis occurs during seed priming, but it was not essential for rapid germination rates (Gurusinghe et al., 1999a, Gurusinghe et al., 1999b). The majority of embryo cells in mature dry seeds are in the G1 stage of cell cycle (Velappan et al., 2017). In Arabidopsis, the embryo growth is initiated by cell expansion during radicle emergence and it also causes the cell cycle activation in the root apical meristem (RAM) (Vázquez-Ramos and de la Paz Sánchez, 2007). It is believed that activity of cell cycle is suppressed at some point during maturation phase and at the same time seed dormancy is induced. However, extent of dormancy may differ in seed lots, which probably could be the reason for their differential response to seed priming treatments. Post-germination cell division is actually initially activated in root meristem cells, which is followed by division in cotyledons and shoot apical meristem (SAM) (Barroco et al., 2005, Masubelele et al., 2005). Endocycle, where nuclei replicate DNA to increase the ploidy level without mitotic divisions is linked with the cell expansion. This process in seeds helps in stimulating germination (Finch-Savage and Bassel, 2016). In aged or deteriorated seed, direct correlation between age and increased time for DNA replication has been reported, which is manifested by delayed MGT and declining seed vigour. Apparently, deteriorated seed takes a longer time for its DNA repair and this delay in turn affect all cell cycle related activities (Vázquez-Ramos and Sánchez, 2003).

Presowing treatments, like seed priming has been reported to augment seed planting value in many crops, but can cause significant reduction in longevity *i.e.* it accelerates the loss of viability over time, thereby causing extensive economic losses in crop species (Dekkers et al., 2015). The molecular mechanism for this loss of viability during storage is still obscure. However, loss of storability in primed seed could be accounted for the accumulation of a higher DNA content, which is a more susceptible site for mutation causing factors, and hence, associated with reduced viability in tomato (Van Pijlen et al., 1996). High vigour in the maize seeds might have been due to enhanced cell cycle activity, owing to auxin stimulation (Lara-Núñez et al., 2008).

## 5 Conclusions

Hydropriming for 30 h duration followed by either surface drying or complete redrying significantly enhanced the seed germination and vigour of maize (*Zea mays* L.) seeds. The vigour index I was found to be significantly correlated with G2/G1 ratio whereas significant negative correlation between G2/G1 ratio and mean germination time was observed. Flow cytometric determination of DNA levels in embryos of fully matured dry maize (*Zea mays* L.) seeds revealed large amounts of 2C DNA signals, indicating that most cells had arrested in the cell cycle at the presynthetic G1 phase of nuclear division. The priming period of 30 h in distilled water considerably enhanced the rate and uniformity of germination in both surface dried and redried treatment strategies. Upon priming, the ratio of 4C:2C increased during the 30 h priming period, though the level in case of redried seeds did not reach the level obtained after hydration in water without drying back. However, the 4C: 2C ratio was constant after redrying the seeds towards the original moisture content, indicating that the chromosomal material in the embryonic cells had stably ceased cell cycle activity at the G2 phase. The results of present study indicate that the beneficial effects of priming on seedling performance are associated with the action of replicative DNA synthetic processes prior to germination. Thus, it was obvious that hydropriming treatment influenced the cell cycle activity in the embryonic tissues of the maize seeds. Moreover, it could also be concluded that an increase in 4C DNA content in the cell during hydropriming of maize cultivars is associated with reduced mean germination time (MGT) and enhanced seed vigour.

## Declaration of Competing Interest

The author declare that there is no conflict of interest.
